# The Pro12Ala Polymorphism of PPAR-*γ* Gene Is Associated with Sepsis Disease Severity and Outcome in Chinese Han Population

**DOI:** 10.1155/2014/701971

**Published:** 2014-07-20

**Authors:** Guoda Ma, Haiyang Wang, Guixi Mo, Lili Cui, You Li, Yiming Shao, Xin Liu, Yuliu Xie, Jia Li, Jiawu Fu, Hua Tao, Bin Zhao, Liangqing Zhang, Keshen Li

**Affiliations:** ^1^Guangdong Key Laboratory of Age-Related Cardiac and Cerebral Diseases, Affiliated Hospital of Guangdong Medical College, Zhanjiang 524001, China; ^2^Department of Vascular Surgery of General Surgery, The First Affiliated Hospital of Harbin Medical University, Harbin 150001, China; ^3^Department of Anesthesiology, Affiliated Hospital of Guangdong Medical College, Zhanjiang 524001, China; ^4^Intensive Care Unit, Affiliated Hospital of Guangdong Medical College, Zhanjiang 524001, China

## Abstract

Peroxisome proliferator-activated receptor-*γ* (PPAR-*γ*) is a ligand-binding nuclear receptor, and its activation plays a prominent role in regulating the inflammatory response. Therefore, PPAR-*γ* has been suggested as a candidate gene for sepsis. In the present study, we investigated the association between the Pro12Ala polymorphism of PPAR-*γ* and sepsis in a Han Chinese population. A total of 308 patients with sepsis and 345 healthy controls were enrolled in this study. Genotyping was performed using the polymerase chain reaction-ligation detection reaction (PCR-LDR) method. No significant differences were detected in the allele and genotype distributions of the PPAR-*γ* Pro12Ala SNP between septic patients and controls (*P* = 0.622 for genotype; *P* = 0.629 for allele). However, stratification by subtypes (sepsis, septic shock, and severe sepsis) revealed a statistically significant difference in the frequency of the Ala allele and Ala-carrier genotype between the patients with the sepsis subtype and the healthy controls (*P* = 0.014 for allele and *P* = 0.012, for genotype). Moreover, significant differences were found in the frequency of the Ala allele and genotype between the sepsis survivors and nonsurvivors (all *P* = 0.002). In the survivors, the PPAR-*γ* Pro12Ala genotype was significantly associated with decreased disease severity and recovery time (all *P* < 0.001). Thus, genetic polymorphism is thought to play a role in the development and outcome of sepsis.

## 1. Introduction

Sepsis is a clinical entity involving a massive systemic inflammatory response, which may result in septic shock, multiple organ system failure, and death [1]. Although the methods of treatment are constantly updated and refined, sepsis and septic shock remain the most prevalent causes of death in intensive care units (ICUs). Recently, peroxisome proliferator-activated receptor gamma (PPAR-*γ*) was described as playing a role in modulating the pathological status of sepsis by regulating energy metabolism, inflammation, and immune cell function [2].

PPAR-*γ*, a member of the nuclear hormone receptor superfamily, is a ligand-binding nuclear receptor whose activation controls the inflammatory response [3]. PPAR-*γ* has been shown to regulate inflammatory status by controlling the differentiation of monocytes and macrophages and by suppressing the expression of inflammatory cytokines, such as tumor necrosis factor alpha (TNF-*α*), interleukin-1 beta (IL-1*β*), inducible cyclooxygenase-2 (COX-2), and other downstream markers of inflammation [3–5]. In animal models of sepsis and septic shock, PPAR-*γ* agonist pretreatment markedly attenuated inflammation compared with controls [6, 7]. Therefore, PPAR-*γ* has been suggested to be beneficial in sepsis. However, PPAR-*γ* induces apoptosis, and the death of immune cells, especially T lymphocytes, is generally considered deleterious [2]. Under these circumstances, a second infection cannot be adequately cleared, leading to septic shock and multiple organ dysfunction syndrome. Because of the profound involvement of PPAR-*γ* in sepsis, we explored the possibility of PPAR-*γ* as a candidate gene for sepsis susceptibility.

The most common functional polymorphism in the* PPAR*-*γ* gene is a CCA-to-GCA missense mutation (rs1801282) in codon 12 of exon B, which results in the replacement of proline 12 with alanine (Pro12Ala) [8] and a reduction in the transcriptional activity of PPAR-*γ* [9]. Previous studies have examined the PPAR-*γ* Pro12Ala polymorphism in a variety of inflammatory diseases, such as atherosclerosis, psoriatic arthritis, inflammatory bowel disease, and multiple sclerosis [10–13], but none have documented an association between this polymorphism and sepsis. Therefore, we conducted a hospital-based case-control study to investigate whether this functional polymorphism of the* PPAR*-*γ* gene affects the risk, disease severity, and outcome of sepsis in a Chinese Han population.

## 2. Materials and Methods 

### 2.1. Participant Recruitment

In this study, all the subjects were recruited from the Department of Emergency and ICU of the Affiliated Hospital of Guangdong Medical College between March 2011 and October 2013. Blood samples were collected from the subjects upon the diagnosis of sepsis, severe sepsis, or septic shock. These patients were diagnosed with sepsis, severe sepsis, or septic shock according to the 1991 ACCP/SCCM Joint Meeting [14] and the diagnostic criteria developed at the 2001 International Sepsis Definition Conference [15]. Patients below 18 years of age or suffering from diabetes, immunological diseases, or malignancies were excluded from this study. A total of 308 patients with sepsis were observed during the ICU stay until death or hospital discharge occurred. The Acute Physiology and Chronic Health Evaluation II (APACHE-II) and Sequential Organ Failure Assessment (SOFA) scores were determined on the day of ICU admission and were used to evaluate illness severity and organ dysfunction/failure, respectively. Based on 28-day survival data, the patients with sepsis were further divided into a survivor group (≥28-day survival) and a nonsurvivor group (<28-day survival). All the patients were observed for 28-day survival, which was calculated from the date of the primary diagnosis of sepsis. Upon admission to the ICU, patients with sepsis underwent the daily collection of physiologic and laboratory data, for example, gender, age, chronic disease status, cause of sepsis, site of infection, chief complications, duration of ICU stay, duration of hospital stay, prognosis, APACHE-II scores, and SOFA scores. Concurrently, 345 healthy Chinese Han individuals were genotyped and functioned as a control population for the genotype analysis. The Ethics Committee of the Affiliated Hospital of Guangdong Medical College approved this study, and informed consent was obtained from the patients and/or their family members.

### 2.2. DNA Extraction and Genotyping

Genomic DNA was isolated from the EDTA blood samples collected from all of the patients and controls using the Blood DNA Kit (Tiangen Biotech, Beijing, China) according to the manufacturer's instructions. For each sample, the PPAR-*γ* Pro12Ala genotype was determined using the polymerase chain reaction-ligation detection reaction (PCR-LDR) method. The PCR primers for Pro12Ala were as follows: forward primer 5′-TGATGTCTTGACTCATGGGTGT-3′ and reverse primer 5′-TACATAAATGCCCCCACGTC-3′. PCR was performed in a Perkin-Elmer GeneAmp PCR System 9600 (Applied Biosystems, USA) in a total reaction volume of 20 *μ*L containing 1 *μ*L of genomic DNA, 2 *μ*L of 1× Taq buffer, 0.4 *μ*L of each primer, 2 *μ*L of dNTPs, 0.3 *μ*L of Qiagen HotStarTaq polymerase (Qiagen, Germany), and 9.8 *μ*L of H_2_O. The amplification parameters were as follows: denaturation at 95°C for 15 min; 35 cycles of denaturation at 94°C for 30 s, annealing at 57°C for 90 s and extension at 72°C for 60 s; and a final extension step at 72°C for 10 min. The probes for LDR were as follows: 5′-P-GTCAATAGGAGAATCTCCCAGAGT -FAM-3′, with a phosphorylated 5′ end and a 6-carboxyXuorescein (FAM)-labeled 3′ end; a C-specific probe, 5′-TGTATCAGTGAAGGAATCGCTTTCTGG-3′; and a G-specific probe, 5′-TTTGTATCAGTGAAGGAATCGCTTTCTGC-3′. A ligation reaction was performed with each PCR product; the final volume of 10 *μ*L contained 2 *μ*L of PCR product, 1 *μ*L of 1× Taq DNA ligase buffer, 1 *μ*L of probe mixture, 2 U of Taq DNA ligase (New England Biolabs, USA), and 6.95 *μ*L of H_2_O. The reaction conditions for LDR were as follows: denaturation at 95°C for 2 min, followed by 30 cycles of 94°C for 15 s and 50°C for 25 s. The fluorescent LDR products were analyzed using an ABI 377 DNA Sequencer (Applied Biosystems, USA).

### 2.3. Statistical Analysis

The statistical analysis was performed with SPSS version 19.0 (SPSS Inc., Chicago, IL, USA). The results for continuous variables with normal distributions are provided as means ± standard deviations (SD). Student's *t*-test was performed to compare means between two groups. The genotype distributions of all the groups were assessed for deviations from Hardy-Weinberg equilibrium. Allele and genotype frequencies were compared using the chi-squared test or Fisher's exact two-tailed tests when appropriate. Kaplan-Meier survival analysis in 28 days was used to explore the mortality differences. The log-rank test was used to evaluate the univariate relationship between the PPAR-*γ* Pro12Ala genotype and clinical outcome. Values of *P* < 0.05 were considered statistically significant.

## 3. Results

### 3.1. Clinical Characteristics

The baseline characteristics and clinical data of all the subjects are shown in Table 1. The average age and sex distribution did not differ significantly between the sepsis and healthy control groups or between the survivor and nonsurvivor groups. The respiratory tract (77.9%), bloodstream (27.6%), and abdomen (35.4%) were the main sites of infection. Gram-negative infections (33.8%) and mixed infections (35.1%) were the primary infection types, while fungal infections accounted for 10.4%. Among the total group of sepsis patients, 45.8% exhibited three or more organ dysfunctions. Severe sepsis accounted for 58.8% of the sepsis patients. The overall 28-day mortality rate of the sepsis patients was 36.5%.

### 3.2. Distributions of Genotypes and Allele Frequencies in Sepsis Patients and Controls

A total of 308 sepsis patients and 345 healthy control subjects were successfully analyzed for the Pro12Ala polymorphism. The genotype and allele frequency distributions of this single-nucleotide polymorphism (SNP) in the patient groups and control groups in our cohort are presented in Table 2. The allele and genotype distributions for the assayed locus, rs1801282, of the patients with sepsis and controls indicated that both groups were in Hardy-Weinberg equilibrium (*P* = 0.536 and 0.449, resp.). The frequencies of the Pro12Pro and Pro12Ala genotypes were 93.18% and 6.82%, respectively, in the case group, and 92.17% and 7.83%, respectively, in the control group. None of the individuals in our study cohort had the Ala12Ala genotype.

### 3.3. Disease Severity and Genotype

We divided the sepsis cases into three subtypes (sepsis, septic shock, and severe sepsis) and investigated the association between the SNP and each subtype. As shown in Table 3, there were significant differences in the genotype distribution and allele frequency between the sepsis subtype and the healthy controls. The frequency of the Ala allele in the sepsis subtype was significantly higher than in the healthy controls (*P* = 0.014, odds ratio (OR) = 3.243, and 95% confidence interval (CI) (1.349–7.796) for allele and *P* = 0.012, OR = 3.585, and 95% CI (1.410–9.111) for genotype). Furthermore, after Benjamini-Hochberg (BH) multiple testing correction was performed, the difference remained significant (P* (corr) = 0.042 for genotype and P* (corr) = 0.042 for allele). There were no significant differences in genotype distribution or allele frequency between the healthy controls and the septic shock or severe sepsis subtype.

We also separated the sepsis cases into two groups, survivors and nonsurvivors, on the basis of the patients' 28-day mortality. Only one patient in the nonsurvivor group had the Pro12Ala genotype. Significant differences in genotype and allele frequencies were found between the survivors and nonsurvivors in the overall group of sepsis patients (*P* = 0.002, P* (corr) = 0.002 for genotype and *P* = 0.002, P* (corr) = 0.002 for allele) (Table 4).

### 3.4. Mortality and Genotype

A total of 112 (36.5%) patients died during hospitalization. The mortality increased with increasing severity of the disease, from 13.3% for sepsis to 32.0% and 51.5% for severe sepsis and septic shock, respectively (*P* < 0.001). The overall group of patients with sepsis (308 cases) was further divided into two groups according to genotype.

**Figure 1:**
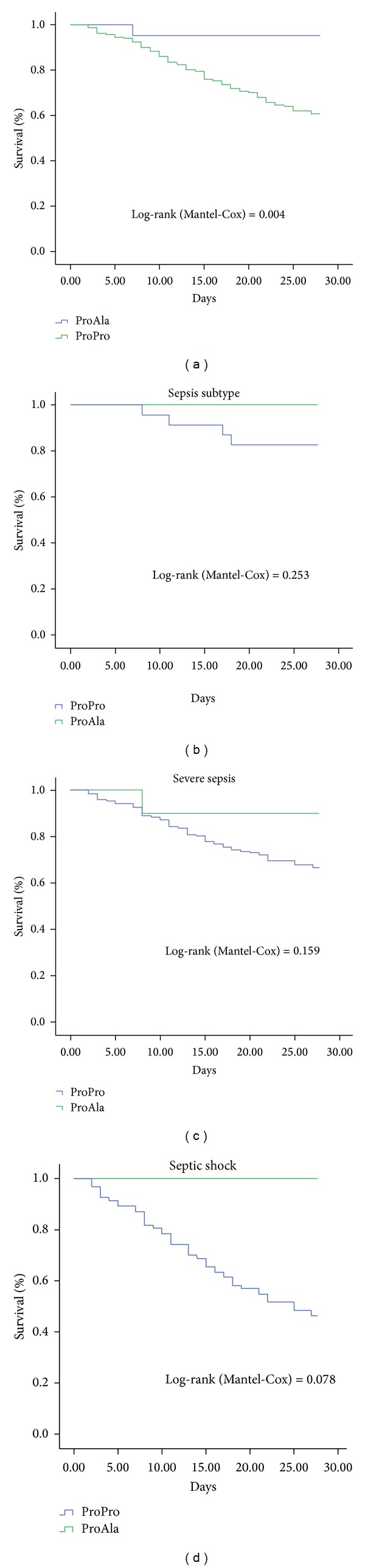
28-day survival rates of patients with sepsis. Kaplan-Meier estimates were used to calculate the probability of 28-day survival according to the PPAR-*γ* Pro12Ala genotype. The carriers of the Pro12Pro genotype had significantly decreased 28-day survival compared with the carriers of the Pro12Ala genotype. (a) Pro12Pro carriers versus Pro12Ala carriers; (b) Pro12Pro carriers versus Pro12Ala carriers in Sepsis subtype subgroup; (c) Pro12Pro carriers versus Pro12Ala carriers in severe sepsis subgroup; (d) Pro12Pro carriers versus Pro12Ala carriers in severe sepsis subgroup in septic shock subgroup.

Based on a log-rank test for trend, the Ala12 allele carriers had significantly increased 28-day survival compared with the Pro12 carriers (*P* = 0.004) (Figure 1(a)). However, stratification by subtypes (sepsis subtype, severe sepsis, and septic shock) did not reveal a statistical difference between the Pro12 and the Ala12 allele carriers in sepsis patients (Figures 1(b), 1(c), and 1(d)). A small trend, although not significant, was observed between the Pro12 and the Ala12 allele carriers in septic shock patients (*P* = 0.078) (Figure 1(d)).

### 3.5. Association Analysis of the PPAR-*γ* Gene Polymorphism and Sepsis Outcome in Survivors

The patients in the survivor group were divided into two groups according to their genotype. There were significant differences between the two groups in the APACHE-II score, SOFA score, duration of ICU stay, and duration of hospital stay. The patients with the Pro12Pro genotype had worse disease severity and increased recovery time compared with the patients with the Pro12Ala genotype (Table 5).

## 4. Discussion

We investigated a common SNP, Pro12Ala, in the PPAR-*γ* gene in 308 patients with clinically defined sepsis and 345 age-matched healthy controls, and we evaluated the effects of this SNP on disease risk and progression. No significant differences were found in the genotype distribution and allele frequency of the PPAR-*γ* Pro12Ala polymorphism between the sepsis patients and healthy controls. However, in the group of survivors, we found that carrying the Ala allele of the Pro12Ala polymorphism was significantly associated with disease severity. Based on our Kaplan-Meier survival analysis, 28-day survival was significantly reduced in individuals with the Pro12Pro genotype compared with the Pro12Ala genotype. The Ala12 carriers had lower disease severity scores, lower mortality, and faster recovery compared with the patients with wild-type genotypes. Our population-based study demonstrated that the Pro12Ala polymorphism allele might not serve as a marker for susceptibility to sepsis but could influence a patient's clinical outcome and risk of dying from sepsis.

PPAR-*γ* is a nuclear receptor expressed in monocytes, macrophages, T cells, endothelial cells, and other cells involved in the progression of sepsis [4, 16–19]. Importantly, compared with control subjects, increases in PPAR-*γ* expression and activity have been reported in T lymphocytes and polymorphonuclear neutrophils isolated from either mice or human patients with sepsis [16, 20, 21]. PPAR-*γ* activation during the onset of sepsis inhibits inflammatory gene expression and can negatively interfere with proinflammatory transcription factor signaling pathways in inflammatory cells, resulting in the prevention of sepsis progression via an attenuation of the hyperinflammatory response. The body diminishes the harmful effects of the late phase of sepsis by producing anti-inflammatory cytokines and enhancing immune paralysis via immune cell apoptosis [22, 23]. Hotchkiss and colleagues showed that the depletion of lymphocytes is the central pathogenic event during sepsis and is a major contributor to poor outcomes following sepsis [22, 24–27]. Compared with the major (Pro) allele, the minor (Ala) allele of PPAR-*γ* is less biochemically active; therefore, we speculated that the improved survival and outcome of Ala carriers might be due to an attenuation of T cell apoptosis. Our results provide strong support for this hypothesis. In addition, the survival advantage conferred by the Ala12 allele during sepsis is also supported by* in vivo* studies of T cell-specific PPAR-*γ* knockout mice and* in vitro* studies of PPAR-*γ* inhibition in T cells using the PPAR-*γ* antagonist GW9662 [16].

To our knowledge, our study is the first to analyze the association between the PPAR-*γ* Pro12Ala polymorphism and sepsis in a Chinese Han population. In this study, the Ala allele of the PPAR-*γ* Pro12Ala polymorphism was associated with significant benefits in the clinical outcome of sepsis. Previous studies have reported that the Ala allele was correlated with lower PPAR-*γ* transcriptional activity and have characterized the PPAR-*γ* Pro12Ala polymorphism in a variety of inflammatory diseases, such as type 2 diabetes mellitus, atherosclerosis, ulcerative colitis, Crohn's disease, psoriatic arthritis, and diabetic nephropathy [10–12, 28, 29]. Most of these studies observed a protective effect in carriers of the Ala allele. Although the present study found no significant differences between the healthy controls and sepsis patients in the distribution of Pro12Ala genotypes or alleles, the sepsis patients in the survivor with the PPAR-*γ* Pro12Ala genotype had milder disease and faster recovery than those with the Pro12Pro genotype.

The Ala12 allele frequency of the Pro12Ala SNP varies widely among continental and ethnic groups; reported Ala12 allele frequencies include 0.15 in a Finnish population, 0.12 in a German population, 0.082 in an Italian population, and 0.034 in a Chinese population [30]. In the context of sepsis, this variation might result in diverse genetic roles of this polymorphism in different populations. Therefore, it will be necessary to confirm our findings in patients of different ethnicities.

In summary, our results reveal that the PPAR-*γ* Pro12Ala polymorphism is not associated with sepsis in the studied Chinese Han population, but the observed genetic difference may be important in influencing clinical outcome.

## Figures and Tables

**Table 1:** Demographic and clinical characteristics of the study subjects in the sepsis and control groups.

Characteristics	Cases (*n* = 308)	Controls (*n* = 345)	*P* value
	*n* (%)	*n* (%)	
Age (years)	62.36 ± 17.17	54.90 ± 15.73	0.412
Male/female, *n*	217/91	222/123	0.097

Organ dysfunction			
One, *n* (%)	48 (15.6)	N.A.	
Two, *n* (%)	89 (28.9)	N.A.	
Three or more, *n* (%)	141 (45.8)	N.A.	

Sepsis status			
Sepsis, *n* (%)	30 (9.7)	N.A.	
Septic shock, *n* (%)	97 (31.5)	N.A.	
Severe sepsis, *n* (%)	181 (58.8)	N.A.	

Source of infection, *n* (%)			
Respiratory tract infection	240 (77.9)	N.A.	
Primary bloodstream infection	85 (27.6)	N.A.	
Wound infection	38 (12.3)	N.A.	
Abdominal infection	109 (35.4)	N.A.	
Urinary tract infection	13 (4.2)	N.A.	
Catheter-associated infection	25 (8.1)	N.A.	
Other	19 (6.2)	N.A.	

Pathogens, *n* (%) (positive blood cultures)			
Gram-negative	104 (33.8)	N.A.	
Gram-positive	51 (16.5)	N.A.	
Mixed Gram-negative and positive	108 (35.1)	N.A.	
Fungus	32 (10.4)	N.A.	
Negative blood cultures	13 (4.2)	N.A.	
APACHE-II score	23.1 ± 4.7	N.A.	
28-day mortality, *n* (%)	36.5	N.A.	

N.A.: not Applicable; APACHE II: Acute Physiology and Chronic Health Evaluation II.

**Table 2:** Distributions of genotypes and allele frequencies in controls and patients with sepsis.

Genotype	All sepsis cases, *n* (%)	Controls, *n* (%)	*P* value	*P* value∗	OR (95% CI)
Total	308	345	0.622	0.629	1.160 (0.642–2.098)
Pro12Pro	287 (93.18)	318 (92.17)			
Pro12Ala	21 (6.82)	27 (7.83)			
Ala12Ala	0 (0)	0 (0)			
Allele					
Pro	595 (96.59)	663 (96.09)	0.629	0.629	1.154 (0.645–2.063)
Ala	21 (3.41)	27 (3.91)			

OR: odds ratio; 95% CI: 95% confidence interval. *False discovery rate adjusted  *P* value for multiple hypothesis testing using the Benjamini-Hochberg method.

**Table 3:** Distributions of genotype and allele frequencies in the sepsis subtypes and healthy controls.

Genotype	Healthy controls *n* (%)	Sepsis (subtype) *n* (%)	Septic shock *n* (%)	Severe sepsis *n* (%)	*P* value			*P* value∗		
					*P*1	*P*2	*P*3	*P*1*	*P*2*	*P*3*
Total	345	30	97	181	0.012	0.264	0.327	0.042	0.336	0.336
Pro12Pro	318 (92.17)	23 (76.67)	93 (95.88)	171 (94.48)						
Pro12Ala	27 (7.83)	7 (23.33)	4 (4.12)	10 (5.52)						
Allele										
Pro	663 (96.09)	53 (88.33)	190 (97.94)	352 (97.24)	0.014	0.273	0.336	0.042	0.336	0.336
Ala	27 (3.91)	7 (11.67)	4 (2.06)	10 (2.76)						

OR: odds ratio; 95% CI: 95% confidence interval. *False discovery rate adjusted  *P* value for multiple hypothesis testing using the Benjamini-Hochberg method. *P*1 and *P*1*: healthy control group versus sepsis group. *P*2 and *P*2*: healthy control group versus septic shock group. *P*3 and *P*3*: healthy control group versus severe sepsis group. Fisher's exact test: *P*1 = 0.012, OR = 3.585, and 95% CI (1.410–9.111) for genotype and *P*1 = 0.014, OR = 3.243, 95% CI (1.349–7.796) for allele.

**Table 4:** Distributions of genotype and allele frequencies among surviving and nonsurviving patients.

Genotype	Survivors *n* (%)	Nonsurvivors *n* (%)	*P* value	*P* value∗	OR (95% CI)
Total	196	112			
Pro12Pro	176 (89.80)	111 (99.11)	0.002	0.002	12.614 (1.669–95.31)
Pro12Ala	20 (10.20)	1 (0.09)			
Allele					
Pro	372 (94.90)	223 (99.55)	0.002	0.002	11.989 (1.598–89.95)
Ala	20 (5.10)	1 (0.45)			

OR: odds ratio; 95% CI: 95% confidence interval. *False discovery rate adjusted  *P* value for multiple hypothesis testing using the Benjamini-Hochberg method.

**Table 5:** Association between genotype and sepsis outcome in the survivor group.

Genotype	*n*	APACHE-II score	SOFA score	ICU stay (days)	Hospital stay (days)
Pro12Pro	176	20.78 ± 3.7	11.21 ± 4.2	18.75 ± 3.9	25.981 ± 7.1
Pro12Ala	20	15.01 ± 3.3	7.05 ± 2.8	12.33 ± 2.4	17.72 ± 5.1
*P* value		0.031	0.029	0.025	0.021
*P* value∗		0.031	0.031	0.031	0.031

*False discovery rate adjusted  *P* value for multiple hypothesis testing using the Benjamini-Hochberg method.
